# Identification of an individualized therapy prognostic signature for head and neck squamous cell carcinoma

**DOI:** 10.1186/s12864-023-09325-1

**Published:** 2023-04-28

**Authors:** Cheng Lin, Yuebing Chen, Jianji Pan, Qiongjiao Lu, Pengjie Ji, Shuiqin Lin, Chunfeng Liu, Shaojun Lin, Meifang Li, Jingfeng Zong

**Affiliations:** 1 Department of Radiation Oncology, Clinical Oncology School of Fujian Medical University, Fujian Cancer Hospital, Fuzhou, 350014 Fujian Province China; 2 Department of Radiation Oncology, Fujian Medical University Xiamen Humanity Hospital, Xiamen, Fujian Province China; 3 Department of Medical Oncology, Clinical Oncology School of Fujian Medical University, Fujian Cancer Hospital, Fuzhou, 350300 Fujian Province China

**Keywords:** Head and neck squamous cell carcinoma, Bioinformatics, Therapeutic response, Radiotherapy sensitivity, Prognosis

## Abstract

**Background:**

Head and neck squamous cell carcinoma (HNSCC) are the most common cancers in the head and neck. Therapeutic response-related genes (TRRGs) are closely associated with carcinogenesis and prognosis in HNSCC. However, the clinical value and prognostic significance of TRRGs are still unclear. We aimed to construct a prognostic risk model to predict therapy response and prognosis in TRRGs-defined subgroups of HNSCC.

**Methods:**

The multiomics data and clinical information of HNSCC patients were downloaded from The Cancer Genome Atlas (TCGA). The profile data GSE65858 and GSE67614 chip was downloaded from public functional genomics data Gene Expression Omnibus (GEO). Based on TCGA-HNSC database, patients were divided into a remission group and a non-remission group according to therapy response, and differentially expressed TRRGs between those two groups were screened. Using Cox regression analysis and Least absolute shrinkage and selection operator (LASSO) analysis, candidate TRRGs that can predict the prognosis of HNSCC were identified and used to construct a TRRGs-based signature and a prognostic nomogram.

**Result:**

A total of 1896 differentially expressed TRRGs were screened, including 1530 upregulated genes and 366 downregulated genes. Then, 206 differently expressed TRRGs that was significantly associated with the survival were chosen using univariate Cox regression analysis. Finally, a total of 20 candidate TRRGs genes were identified by LASSO analysis to establish a signature for risk prediction, and the risk score of each patient was calculated. Patients were divided into a high-risk group (Risk-H) and a low-risk group (Risk-L) based on the risk score. Results showed that the Risk-L patients had better overall survival (OS) than Risk-H patients. Receiver operating characteristic (ROC) curve analysis revealed great predictive performance for 1-, 3-, and 5-year OS in TCGA-HNSC and GEO databases. Moreover, for patients treated with post-operative radiotherapy, Risk-L patients had longer OS and lower recurrence than Risk-H patients. The nomogram involves risk score and other clinical factors had good performance in predicting survival probability.

**Conclusions:**

The proposed risk prognostic signature and Nomogram based on TRRGs are novel promising tools for predicting therapy response and overall survival in HNSCC patients.

**Supplementary Information:**

The online version contains supplementary material available at 10.1186/s12864-023-09325-1.

## Background

Head and neck cancer has a yearly incidence of over 800,000 new cases worldwide, with a mortality rate of more than 7%. Head and neck squamous cell carcinoma (HNSCC) accounts for 90% of head and neck cancers [1]. HNSCC are related to continuous exposure to tobacco, tobacco products, and alcohol. In recent years, an increasing proportion of HNSCC caused by human papillomavirus (HPV) infection has been reported [2, 3]. Patients with HPV-associated HNSCC are younger and more responsive to chemotherapy and radiotherapy [4–6]. However, HNSCC is prone to regional lymph node metastasis and distant metastasis, leading to a relatively poor prognosis [7].

HNSCC is treated with a comprehensive treatment strategy, including radiotherapy (RT), surgery, chemotherapy, and targeted therapy et al. However, patients who have significant benefit from therapy remain unknown. Studies have shown that genetic alterations and immune characteristics in the tumor microenvironment (TME) are closely associated with therapy sensitivity and effectiveness [8–10]. Therefore, the identification of potential biomarkers is of great importance in predicting the benefit of therapy, especially radiotherapy, enabling precision and individualization of treatment in HNSCC patients.

In this study, we sought to explore a prognostic risk biomarker for predicting therapeutic efficacy and prognosis in HNSCC. We focused on differentially expressed therapeutic response-related genes (TRRGs) between remission and non-remission patients by bioinformatics. We found that the prognostic risk signature and Nomogram based on TRRGs was an effective tool for predicting therapy response and prognosis, which is of great importance for individualized radiotherapy in HNSCC.

## Materials and methods

### Clinical samples and data collection

Data of The Cancer Genome Atlas (TCGA)-HNSC was from University of North Carolina TCGA genome characterization center and downloaded from website (https://tcga-xena-hub.s3.us-east-1.amazonaws.com/download/TCGA.HNSC.sampleMap%2FHiSeqV2_exon.gz). The GSE65858 Chip dataset was from Wichmann G’ study [11], and downloaded from website (https://www.ncbi.nlm.nih.gov/geo/query/acc.cgi?acc=GSE65858). The GSE67614 Chip dataset was from Ding L’ study [12], and downloaded from website (https://www.ncbi.nlm.nih.gov/geo/query/acc.cgi?acc=GSE67614). mRNA expression profiles and clinical information related to HNSCC are publicly available. Therefore, this study does not need the ethical approval of the local ethics committee. The prognosis information of gene protein was downloaded from the Human Protein Atlas (HPA) database (https://www.proteinatlas.org/). Immunotherapy data were obtained from the immunotherapy cohort data (29,443,960) of urothelial carcinoma, which was stored in the “Imvigor210CoreBiologies” R package.

### Sample grouping and differentially expressed therapeutic response-related genes (TRRGs) analysis

Patients in TCGA-HNSC were divided into two groups based on their response to therapy. Complete response (CR) or partial response (PR) was set as the remission group. Stable disease (SD) or progressive disease (PD) was set as the non-remission group. Differentially expressed TRRGs between those two groups were analyzed using the “limma” R package. *p* value < 0. 05 and |Fold Change|> 1.5 were chosen as the cut-off values for differential expressed TRRGs analysis. The survival difference was analyzed by Kaplan‒Meier analysis. Multiple testing was not considered since the ranking of the TRRGs in the log-rank test was our focus.

### Functional enrichment analysis

To explore the potential biological functions of differentially expressed TRRGs, Gene Ontology (GO) functions and Kyoto Encyclopedia of Genes and Genomes (KEGG) pathway enrichment analyses were performed by Metascape (https://metascape.org). The formal permission of using KEGG and GO was obtained from Kanehisa laboratories[13].

### The mutation types and immune infiltration between the two groups

The mutation type maps of remission and non-remission groups were drawn using the “ComplexHeatmap” R package. The proportion of immune cells was calculated by CIBERSORT software (https://cibersort.Stanford.Edu/), and the infiltration proportion of 22 immune cells in the tumor expression profile was downloaded from PRECOG (http://precog.Stanford.Edu/) and compared by the Wilcoxon rank sum test.

### Construction of the TRRGs signature

First, univariate Cox regression analysis was used to screen 206 TRRGs that were significantly related to overall survival (OS). Log rank *p* < 0.05 was set as the threshold. Then, 20 TRRGs were obtained using Least absolute shrinkage and selection operator (LASSO) regression analysis, which was useful to establish the most feasible and predictable TRRGs signature. Finally, a prognostic risk model was established based on the 20 TRRGs. The risk score of each patient relied on the therapy response and the regression coefficient obtained from the LASSO regression analysis. The risk score model was constructed by the following formula:

$${{\varvec{R}}{\varvec{i}}{\varvec{s}}{\varvec{k}}{\varvec{S}}{\varvec{c}}{\varvec{o}}{\varvec{r}}{\varvec{e}}}_{{\varvec{i}}}=\sum_{{\varvec{j}}=1}^{{\varvec{n}}}{{\varvec{e}}{\varvec{x}}{\varvec{p}}}_{{\varvec{j}}{\varvec{i}}}\times {{\varvec{\theta}}}_{{\varvec{j}}}$$

where *i* is the number of samples, *j* is the name of TRRGs, *exp* is the expression of the corresponding genes, and *θ* is the regression coefficient of TRRGs in the multivariate Cox regression analysis. Wu and Ren’ articles and their methods to accommodate strong correlations among features are also considered[14, 15].

### Verification and evaluation the prognostic value of the TRRGs signature

To verify the characteristics of the TRRGs signature, we divided the patients into a high-risk group (Risk-H) and a low-risk group (Risk-L) by taking the average risk score value as the cut-off, which was further confirmed by Kaplan‒Meier analysis. A time-dependent receiver operating characteristic (ROC) curve was conducted to estimate the sensitivity and specificity of each risk group based on the area under the curve (AUC) value. We further used the GSE65858 dataset as a validation cohort to evaluate the prognostic value of the TRRGs signature. Univariate and multivariate Cox regression analyses were performed to assess the prognostic capability of the TRRGs-based risk signature for HNSCC patients.

### Gene set enrichment analysis (GSEA) and construction of the nomogram

The expression and prognosis of HNSC-related model genes were queried from the HPA database (https://www.proteinatlas.org/). The differences in tumor mutation burden (TMB), immune checkpoint expression, stromal score, immune score, tumor purity, and immune cell proportion between the Risk-H and Risk-L groups were analyzed by the Wilcoxon test. The signaling pathways involved in TRRGs were determined by gene set enrichment analysis (GSEA). A nomogram based on the results of Cox regression analysis was established to predict the survival rate.

### Statistical analysis

All the above statistical analysis was computed by R software. *p*-value < 0.05 (two-sided) was used as the statistically significant threshold. The survival difference between groups was analyzed by Kaplan‒Meier analysis. For the significance analysis between different characteristics, the Wilcoxon test was used to compare the differences between the two groups of samples, and the Kruskal test was used to compare the differences between multiple groups. Other statistical methods and algorithms used in this article are described in the corresponding steps.

## Results

### Identification of differentially expressed therapeutic response-related genes (TRRGs)

**Fig. 1:**
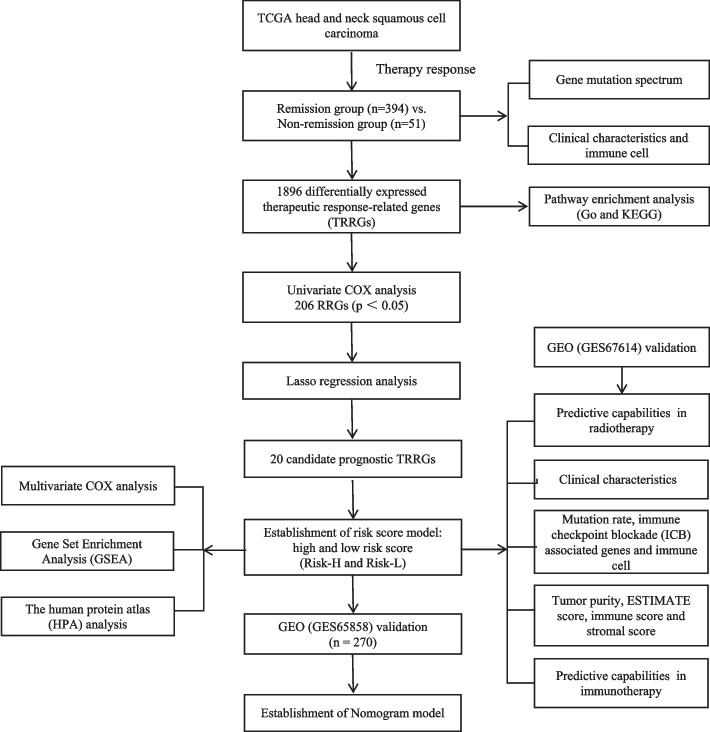
Complete workflow of our research. “n” denotes sample size. “*p* < 0.05” denotes the statistically significant threshold

**Fig. 2:**
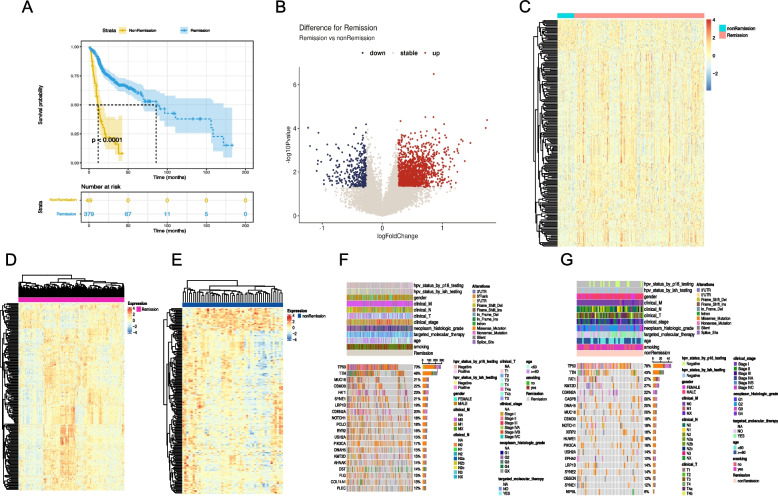
Differentially expressed therapeutic response-related genes (TRRGs) between the remission and non-remission groups in TCGA-HNSC training set data. **A** Survival difference between remission and non-remission groups by Kaplan‒Meier analysis. **B** Volcano plot of 1896 differentially expressed TRRGs, of which 1530 were upregulated and 366 were downregulated. **C-E** Heatmap of the top 200 TRRGs ranked by log fold-change values in remission and non-remission groups. **F**, **G** Mutation types and rates in remission and non-remission groups

The workflow of the study is shown in Fig. 1. For the TCGA-HNSC training set dataset, 445 patients who received therapy and had survival information were enrolled. Patients were divided into a remission group (CR/PR) and a non-remission group (SD/PD) according to therapy response, which was further confirmed by Kaplan‒Meier analysis (Fig. 2A). The remission group and non-remission group contained 394 and 51 patients, respectively. A total of 1896 differentially expressed TRRGs were obtained (Supplemental Table 1), of which 1530 were upregulated and 366 were downregulated (Fig. 2B). The top 200 differentially expressed TRRGs are ranked by log fold-change (FC) values (Fig. 2C), and the distinct gene expression patterns in remission (Fig. 2D) and non-remission groups (Fig. 2E) are also displayed. TP53 and TTN are genes with the highest mutation rates in both groups. The mutation rates of PCLO, RYR2, and AHNAK were significantly higher in the remission group (Fig. 2F), while the mutation rates of CASP8, DNAH5, and XIRP2 were higher in the non-remission group (Fig. 2G). Of note, differentially expressed TRRGs were enriched in tube morphogenesis, chemotaxis, regulation of cell adhesion, etc., using GO and KEGG analysis (Fig. S1).

**Fig. 3:**
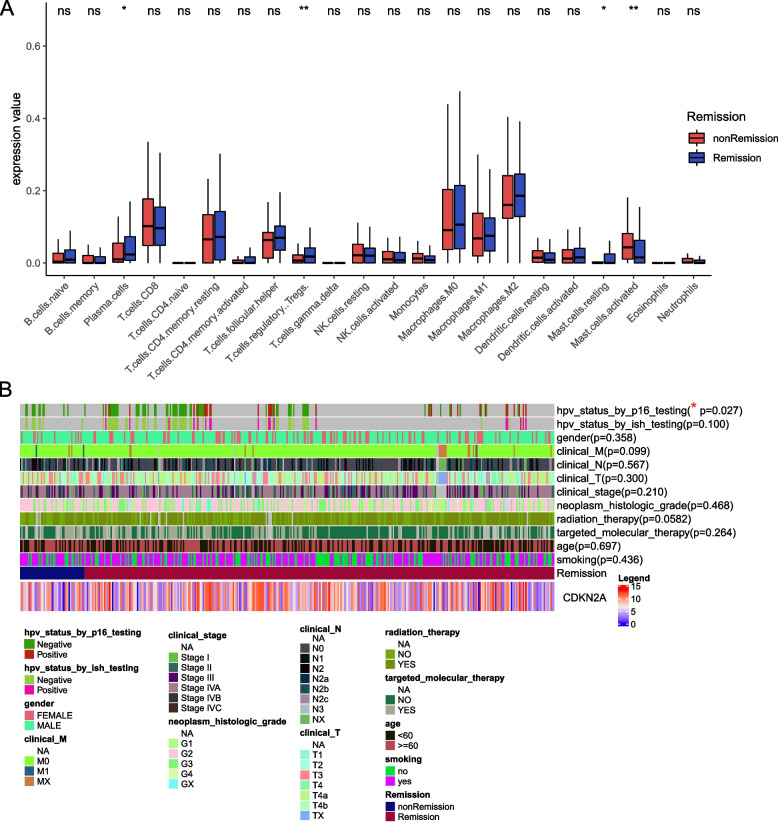
Differences in infiltrating immune cells and clinical characteristics between the remission and non-remission groups. **A** Differences in infiltrating immune cells. **B** Differences in clinical characteristics. ns, not significant; *, *p* < 0.05; **, *p* < 0.01

To address the potential underlying mechanisms that may affect therapy response, clinical characteristics and infiltrating immune cells were investigated. We found that HPV status by p16 testing was significantly associated with improved therapy sensitivity (Fig. 3A). Besides, data showed that plasma cells, T regulatory cells and resting mast cells were higher in the remission group, while activated mast cells were lower in the non-remission group (Fig. 3B).

### Construction of TRRGs signature with LASSO regression analysis

**Fig. 4:**
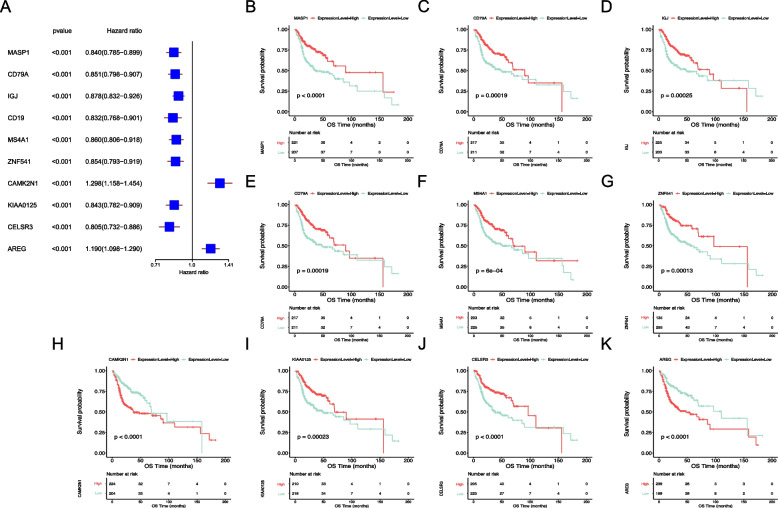
Survival analysis of the top 10 significantly prognostic TRRGs. **A** The random forest of the top 10 TRRGs by univariate Cox analysis. **B**-**K** Kaplan‒Meier analysis of the top 10 TRRGs

To determine the independent prognostic TRRGs, 206 genes associated with OS were chosen and identified using univariate Cox regression (Supplemental Table 2). The top 10 prognostic TRRGs and their prognostic values involved in OS are shown (Fig. 4A). Of the top 10 TRRGs, MK2N1 and AREG were significantly associated with worse outcomes, while the MASP1, CD79A, IGJ, CD19, MS4A1, ZNF541, KIAA0125, and CELSR3 genes showed the opposite trend (Fig. 4B-K). We found that AREG, E1CAM, and GPRC5D had prognostic efficacy in the Human Protein Atlas (HPA) (Fig. S2 A-C), while their mRNA expression was not significantly different between cancer and normal tissues in the TCGA-HNSC database (Fig. S2 D-F).

**Fig. 5:**
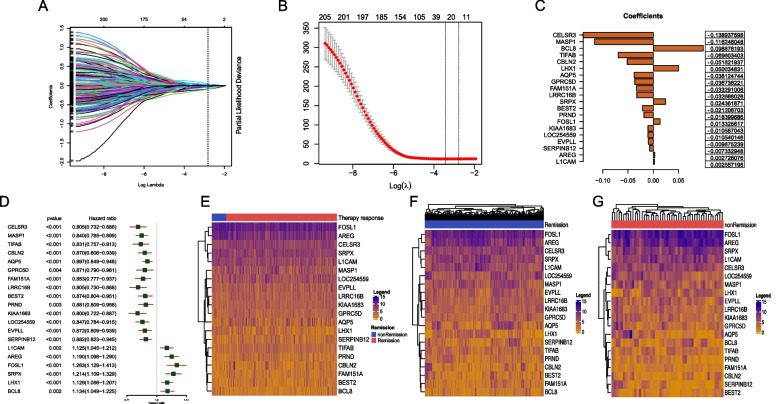
Using LASSO regression analysis to construct the most predictable TRRGs signature. **A** LASSO coefficients distribution of 206 significantly prognostic TRRGs. **B** Tuning parameter (λ) selection cross‐validation error curve. Vertical lines were drawn at the optimal values. **C** Regression coefficient corresponding to the 20 screened TRRGs are shown. A larger absolute value of the coefficient represents a higher correlation. **D** Random Forest of 20 TRRGs. **E** Heatmap of the 20 TRRGs between remission and non-remission groups. **F-G** Different gene expression between remission and non-remission groups

To explore robust TRRGs and potentially prognostic models, TRRGs were further verified by LASSO regression (Fig. 5A). The data showed that with the increase in log lambda, the number of independent coefficients tended to 0 to lower the survival probability (Fig. 5B). The regression coefficient and Cox analysis of the 20 TRRGs were analyzed (Fig. 5C, D). Heatmap further displayed 20 candidate TRRGs (Fig. 5E), and distinct TRRGs patterns in the remission group (Fig. 5F) and non-remission groups (Fig. 5G).

### Validation of the prognostic value of signature

**Fig. 6:**
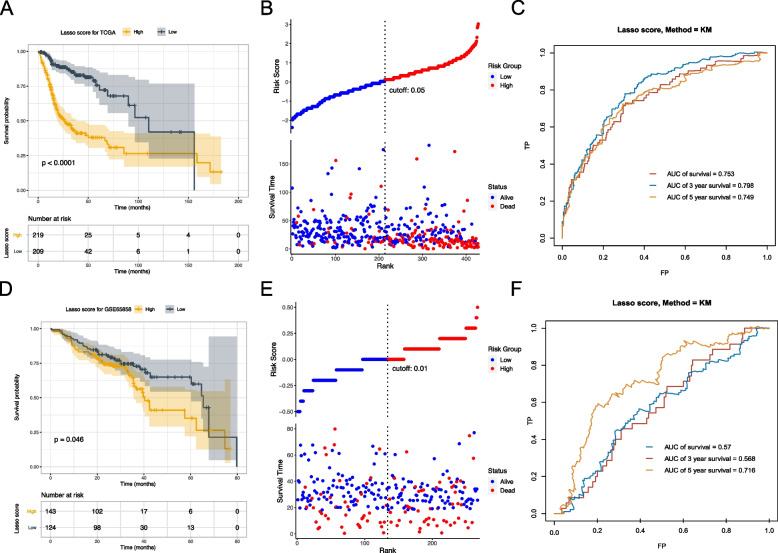
Validation of the prognostic risk model in the TCGA-HNSC cohort (training set) and GSE65858 cohort (validation set). **A**, **D** Survival curves for HNSCC patients with high- and the low-risk score. **B**, **E** Survival status of patients with high- and the low-risk score. **C**, **F** Receiver operating characteristic (ROC) curves for 1-, 3- and 5-year overall survival in HNSCC patients. Up panels are TCGA-HNSC cohort and lower panels are GSE65858 cohort

For guiding a more accurate treatment strategy, a risk prognostic model was constructed based on the 20 candidate TRRGs, then risk score of each sample was calculated (Supplemental Tables 3 and 4). Patients with HNSCC were divided into a high-risk (Risk-H) group and a low-risk (Risk-L) group according to the average risk score of all samples. Next, the TCGA-HNSC cohort (training set) and GSE65858 cohort (validation set) were used to demonstrate the value of the model. Kaplan‒Meier analysis showed that Risk-H was associated with worse OS than Risk-L both in the TCGA-HNSC cohort (*p* < 0.0001) and in the GEO cohort (Fig. 6A, D). The risk score distribution analyses and survival status also illustrated a higher risk of death in the Risk-H group (Fig. 6B, E). The AUCs for 1-, 3-, and 5-years were 0.753, 0.798, and 0.749 in the TCGA-HNSC cohort and 0.570, 0.568, and 0.716 in the GEO cohort, respectively (Fig. 6C, F).

**Fig. 7:**
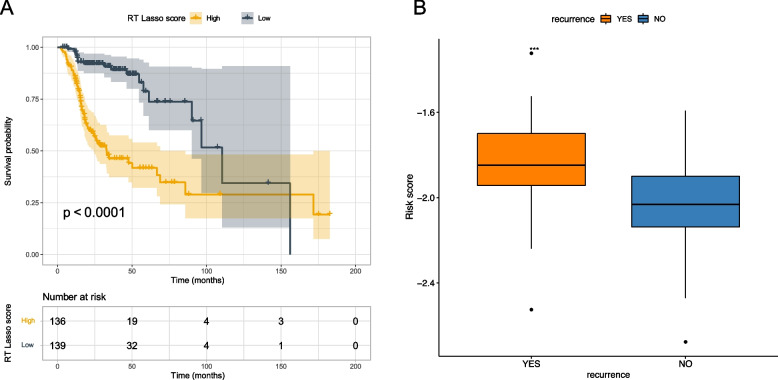
Evaluation of the prognostic value in HNSCC patients who received radiotherapy. **A** Kaplan‒Meier curves based on the TCGA-HNSC dataset. **B** Risk score for recurrent and non-recurrent groups in the GSE65858 cohort

As radiotherapy is a crucial therapy in the management of HNSCC. To further evaluate the value of our prognostic model in HNSCC patients treated with radiotherapy, 275 patients from TCGA-HNSC were included. Data showed that Risk-L patients had better OS than Risk-H patients (Fig. 7A). What’s more, for patients treated with post-operative radiotherapy, Risk-L patients had a lower recurrence than Risk-H patients in the GSE67614 database from GEO (Fig. 7B, Supplemental Table 5). Above data indicating that risk score of TRRGs-based signature was a promising predictive factor for radiotherapy efficacy for HNSCC patients.

Taken together, the risk prognostic signature based on TRRGs was a potential indicator for prognosis and radiation sensitivity in HNSCC.

### Correlations between risk score and clinical characteristics

**Fig. 8:**
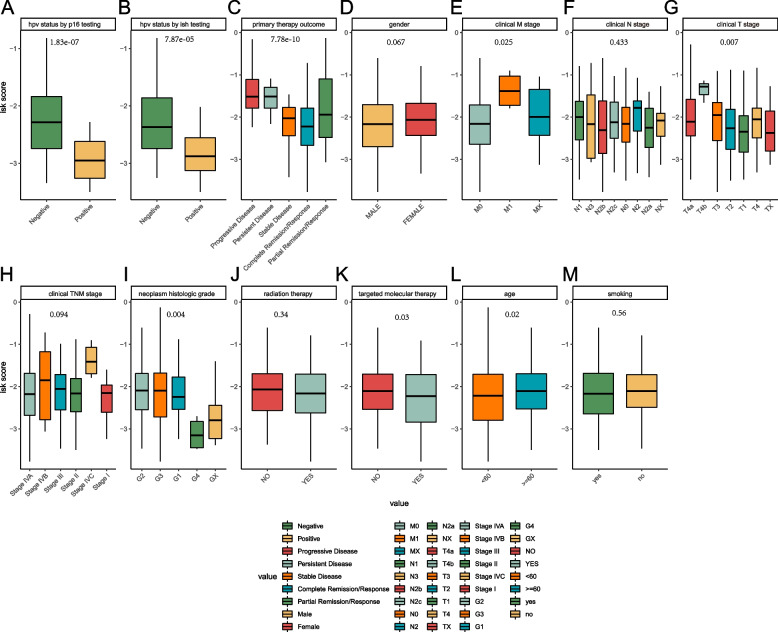
Correlation analysis between risk score and clinical characteristics. The Wilcoxon test was used for double terms and the Kruskal test for multiple terms

To study the relationships between risk score and clinical features, the Wilcoxon test and Kruskal test were used. We found that the risk score was associated with HPV status, primary therapy outcome, clinical M, clinical T, neoplasm histologic grade, targeted molecular therapy, and age. No significance was found among the risk score and gender, clinical N, clinical TNM stage, radiation therapy and smoking (Fig. 8).

### Relationships between risk score and immune status and signaling pathways

**Fig. 9:**
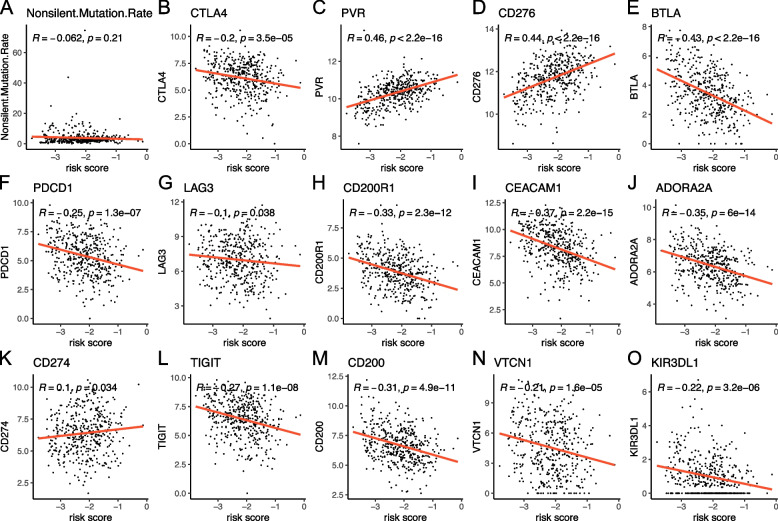
Associations of risk score with tumor mutational burden and immune checkpoint genes. **A** Risk score did not correlate strongly with tumor mutational burden. **B-O** Risk score was significantly associated with multiple immune checkpoint genes

As we described above, the distribution of infiltrating immune cells is significantly different in radiosensitive and resistant HNSCC. To further address the underlying mechanisms between risk score and immune status, tumor mutational burden (TMB), immune checkpoint blockade (ICB)-associated genes, immune score, matrix score, tumor purity and proportion of immune cells were analyzed. We found that there was no significant correlation between risk score and tumor mutational burden (Fig. 9A), while it was significantly associated with numerous immune checkpoint genes. Risk score was positively associated with PVR, CD276, CD274, and was negatively associated with PDCD1, CTLA4, LAG3, etc. (Fig. 9B-O). However, the risk score and nonsilent mutation rates, LAG3, HAVCR2, CD80, CD86, and LGALS3 were not significantly correlated (Fig. S3).

**Fig. 10:**
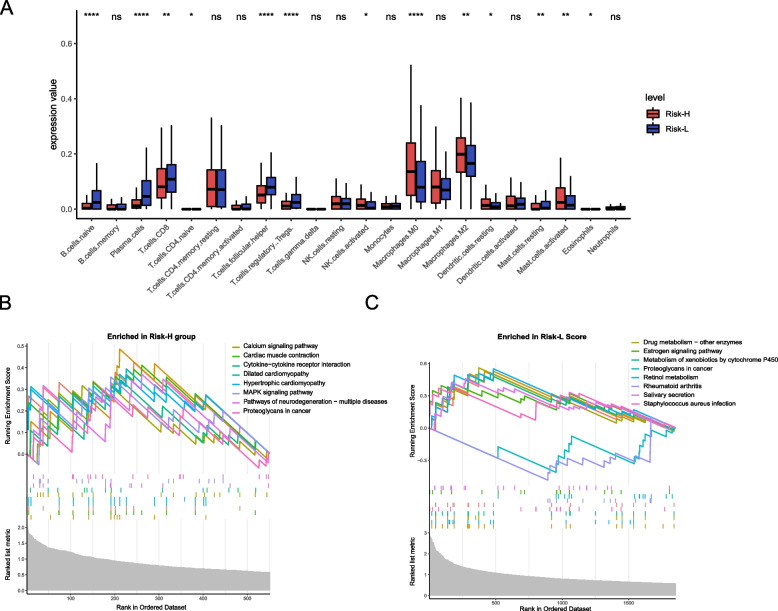
Immune and molecular characteristics of different risk score subgroups. **A** Immune cells between the high-risk score (Risk-H) and low-risk score (Risk-L) groups. **B** Gene sets enriched in the Risk-H group. **C** Gene sets enriched in the Risk-L group. ns, not significant, *, *p* < 0.05, **, *p* < 0.01, ***, *p* < 0.001, ****, *p* < 0.0001)

No significant difference was found among the immune score, matrix score, tumor purity and risk score. However, M0 macrophages, M2 macrophages, activated mast cells, and resting natural killer (NK) cells were more abundant in Risk-H patients, while naive B cells, plasma cells, CD8 T cells, T follicular helper (Tfh) cells, regulatory T cells (Tregs) and resting mast cells were enriched in Risk-L patients (Fig. 10A). These findings suggest that the risk score may be a potential biomarker for immunotherapy response.

Gene set enrichment analysis (GSEA) was conducted to determine the gene sets and pathways in different risk scores. Data showed that Gene sets of Risk-H were enriched in the calcium and MAPK signaling pathway, cytokine‒cytokine receptor interaction and proteoglycans in cancer. (Fig. 10B, C). Gene sets of Risk-L were enriched in drug and retinol metabolism, metabolism of xenobiotics by cytochrome P45, etc.

### Predictive capabilities of risk score in immunotherapy

**Fig. 11:**
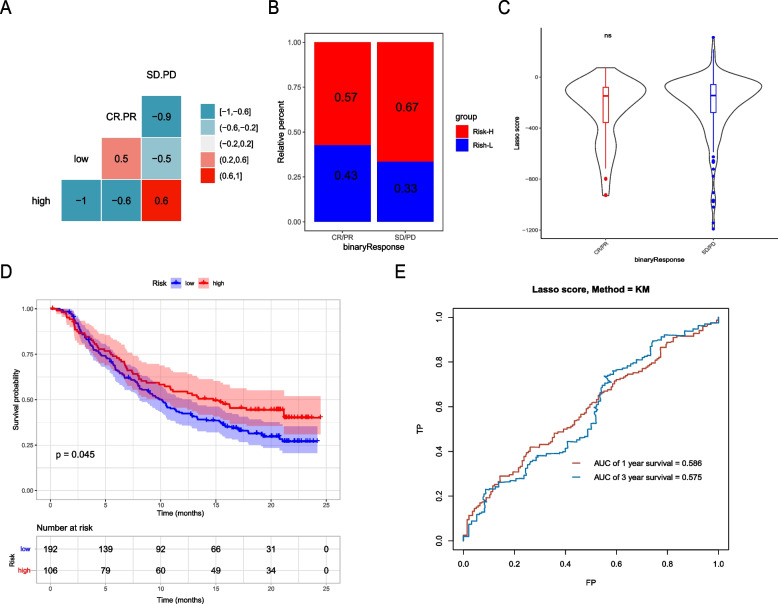
Verified the predictive efficacy of the risk score based on the data from the IMvigor210 cohort. **A** Correlation map of response (complete response / partial response) and nonresponse (stable disease / progressive disease) to immunotherapy in different risk score groups. **B** Relative percent of response and nonresponse to immunotherapy in the Risk-H and Risk-L groups. **C** Comparison of the difference between response and nonresponse groups. **D** Kaplan–Meier curves of overall survival time of the high- and low-risk score groups in the metastatic urothelial carcinoma (mUC) sample. **E** The survival ROC curves of risk score in the mUC sample

As the risk score is somewhat related to immune status, we investigated whether it could predict patients’ response to immunotherapy. The IMvigor210 cohort is an open-label, multicenter, single-arm phase II clinical study that evaluates the safety and efficacy of atezolizumab in patients with metastatic urothelial carcinoma. We found that nonresponse (SD/PD) to immunotherapy was positively correlated with Risk-H (*r* = 0.6, *p* < 0.001), while the response was closely correlated with Risk-L (*r* = 0.5, *p* < 0. 001) (Fig. 11A, B). However, the risk score was not significantly different between the response and nonresponse groups (Fig. 11C). Of note, Risk-H had better OS than Risk-L (*p* = 0.045, Fig. 11D). The AUCs of the risk scores for 1 year and 3 years were 0.586 and 0.575, respectively (Fig. 11E).

### Establishment of the nomogram model

**Fig. 12:**
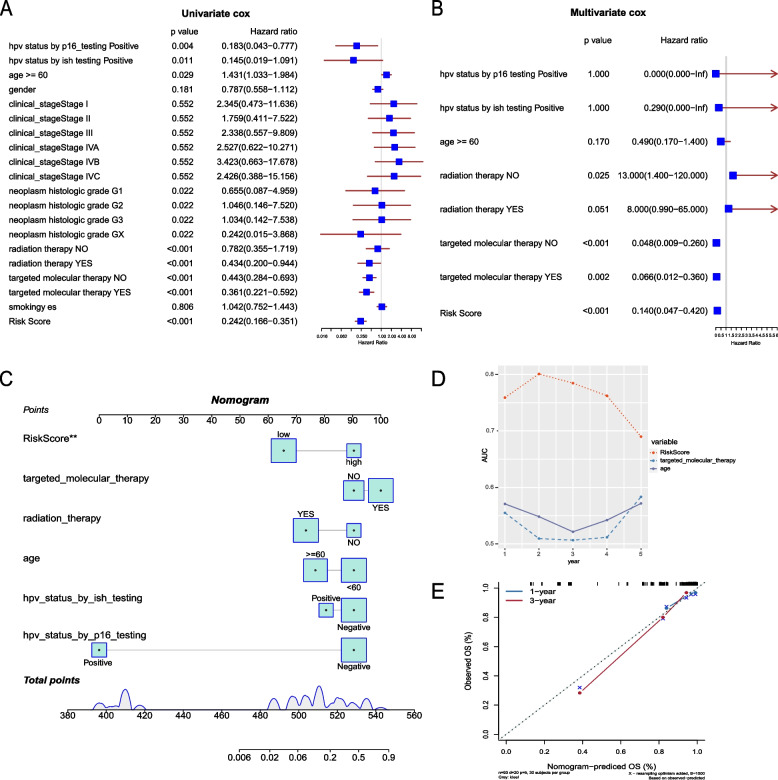
Establishment of the nomogram model. **A**, **B** Univariate and multivariate Cox analyses of clinical features and risk score. **C** Nomogram model of overall survival based on Cox regression analysis. **D** AUC of the risk score, targeted molecular therapy and age. **E** Calibration curves for predicting overall survival at 1 and 3 years

Univariate and multivariate Cox analysis showed that risk score was significantly related to survival (Fig. 12A, B), indicating that the risk score model based on TRRGs was an independent prognostic biomarker in the clinical practice of HNSCC.

To better predict the prognosis of HNSCC, a nomogram was established using five independent prognostic variables based on Cox regression analysis (Fig. 12C). The nomogram showed that the risk score had the highest AUC, followed by age and targeted molecular therapy (Fig. 12D). The calibration curve was then drawn to evaluate the prediction accuracy of the nomogram. The calibration curves of 1 and 3 total OS rates were close to the standard line, suggesting that the nomogram has good performance in predicting survival probability (Fig. 12E).

## Discussion

With the development of the new TNM staging system, transoral robotic surgery, intensity-modulated radiotherapy and immunotherapy, the survival of HNSCC has been greatly improved [16]. Radiotherapy is an indispensable treatment, especially for early-stage and locally advanced disease[17, 18]. However, the prognosis remains poor, as a substantial portion of HNSCC is resistant to radiotherapy[19, 20]. In this study, we conducted a comprehensive bioinformatics analysis to explore a potential biomarker for predicting therapy response and prognosis based on differentially expressed TRRGs. Our data suggested that TRRGs were significantly associated with therapy response, especially radiotherapy. More importantly, the risk score and nomogram model based on TRRGs were promising biomarkers for therapy response and prognosis in HNSCC. Additionally, risk score may be a prognostic biomarker for immunotherapy.

There are various factors that affect the sensitivity of radiotherapy in HNSCC [21–29], including genes, cytokeratin, miRNA, lncRNA, tumor molecular subtypes and anticancer drugs. Increasing prognostic models, based on gene-based signatures, risk score or mutations of key genetic abnormalities, have been explored to predict the response to radiotherapy [30–32]. Fortunately, we observed key differentially expressed TRRGs in HNSCC that affected patients’ response to radiotherapy. We found that CELSR3, AREG and MASP1 were among the top 10 differentially expressed TRRGs and were chosen to construct the risk score model. Of note, high expression of CELSR3 and MASP1 was significantly associated with superior OS, while high expression of AREG was just the opposite in HNSCC. AREG is a predictive biomarker in the treatment of colorectal cancer [33] and promotes the progression of cancers via the AREG/EGFR pathway in pancreatic cancer, breast cancer and ovarian cancer [34–36], suggesting that AREG may be an attractive target in HNSCC. However, CELSR3 has been reported to be highly expressed in hepatocellular carcinoma, prostate cancer, and lung adenocarcinoma and indicates a poor prognosis [37–40]. MASP1 is related to immune cell infiltration in head and neck cancer [41, 42] and could be a candidate target gene in lung cancer and cervical cancer [43, 44]. However, the functions of CELSR3, MASP1 and AREG remains unclear in HNSCC, and the mechanisms how these TRRGs affect the radiotherapy sensitivity and prognosis of HNSCC need to be explored in the future.

Currently, emerging data have demonstrated that HPV + oropharyngeal cancer (OPC) is more sensitive to radiotherapy and has prolonged OS and a reduced risk of death compared to HPV- OPC [45, 46]. HPV status tested by p16 was a reliable and surrogate marker for HPV infection in HPSCC [47]. Consistent with the above studies, our study found that the HPV-positive rate was remarkably higher in the remission group than in the non-remission group, indicating that HPV status was a biomarker for radiotherapy sensitivity. Additionally, the risk score was closely associated with clinical characteristics. Univariable and multivariable analyses suggested that HPV status and risk score were independent prognostic factors in HNSCC. Therefore, the HPV status test is strongly recommended before treatment, and the risk score is also a valuable biomarker, which remains to be confirmed in clinical practice.

To further address the mechanisms that affect therapy response, patients’ immune cells, TMB, ICB-associated genes, etc., and their correlations with risk score were explored. The subtypes of immune cells in the tumor microenvironment varies between the Risk-H and Risk-L groups. We found that CD8 T cells, Tfh cells, and naïve B cells were more abundant in the Risk-L group, indicating an immune hot microenvironment. M2 macrophages were more common in the Risk-H group. M2 macrophages have been proven to be related to chronic inflammation and promote tumor growth and metastases [48]. TMB has served as a valuable biomarker for predicting immune checkpoint inhibitor (ICI) efficacy [49]. In this study, we found that the risk score was not significantly associated with TMB, while it was significantly associated with multiple ICB-associated genes, including CD 274 and CTLA4 LAG3, suggesting that the tumor immune microenvironment (TIME) and ICB-associated genes were closely associated with radiotherapy sensitivity. Of note, the risk score may be a potential tool to predict the immune response and prognosis of patients with metastatic urothelial carcinoma by analyzing the IMvigor210 cohort. Due to the heterogeneity and complexity of the TIME, only a small number of HNSCC patients benefit from immunotherapy. Thus, more studies need to be performed to explain why and how the TIME affects radiotherapy sensitivity. In addition, the relationships between the risk score and immune response remain to be seen. Taken together, our study provides a new idea for exploring the interactions between radiotherapy, immunotherapy, and tumor immune microenvironments in HNSCC.

Last, we found that the nomogram established using the risk score and other clinical features, including age, HPV status, radiation therapy and targeted molecular therapy, had good prognostic efficacy in predicting survival in HNSCC. Nomograms that predict patients’ response to therapy and prognosis in cancers are increasing [50, 51]. Of note, nomograms need to be confirmed in multicenter clinical trials.

Although we identified candidate TRRGs in a large sample through bioinformatics technology, some of the limitations of this study are noteworthy. First, the primary source of clinical features in our dataset is the TCGA-HNSC database. Most of the patients were from North America, so we should be very careful to extend our findings to other geographic and ethnic groups. Second, few clinical characteristics, like p16 status and upfront surgery, were not complete, which may affect the analysis to some extent. Third, our study found 20 TRRGs that are significantly related to the radiotherapy sensitivity and prognosis of patients with HNSCC, while the biological mechanisms are not well understood. Therefore, further studies need to be conducted to confirm our findings.

## Conclusion

In summary, TRRGs play an important role in HNSCC. A risk prognostic signature and Nomogram based on TRRGs is a promising biomarker for predicting therapy response in HNSCC. The underlying regulatory mechanisms of TRRGs may be related to immune cells, ICB-associated genes and the TIME.

## Supplementary Information

**Additional file 1:** **Supplementary Table 1.** A total of 1896 differentially expressed therapeutic response-related genes (TRRGs) in TCGA-HNSC.

**Additional file 2:** **Supplementary Table 2.** There were 206 differentially expressed TRRGs associated with OS.

**Additional file 3:** **Supplementary Table 3. **Risk score for each HNSCC patients in TCGA-HNSC cohorts.

**Additional file 4:** **Supplementary Table 4. **Risk score for each HNSCC patients in GSE65858 cohorts.

**Additional file 5:** **Supplemental Table 5.** The clinical characteristic of HNSCC patientswho received radiotherapy in GSE67614 database.

**Additional file 6:** **Fig. S1.** Functional enrichment and network analysis in the TCGA-HNSC dataset. A Heatmap of the top 20 functional enrichment terms by Gene Ontology (GO) and Kyoto Encyclopedia of Genes and Genomes (KEGG) analysis based on differentially expressed therapeutic response-related genes (TRRGs). B Protein-protein interaction subnetworks of 20 functional enrichment terms.

**Additional file 7:** **Fig. S2** Expression information of AREG, L1CAM and GPRC5D. A-C Kaplan Meier analysis showed that AREG, L1CAM and GPRC5D had a good prognostic efficacy in the Human Protein Atlas (HPA) database. D-F mRNA expression of AREG, L1CAM and GPRC5D was not significantly different between cancer and normal tissues in the TCGA database.

**Additional file 8:** **Fig. S3.** Relationships between the risk score and immune checkpoint blockade (ICB)-associated genes and mutation rate. A ICB-associated genes and nonsilent mutation rate in the Risk-H and Risk-L groups. B Tumor purity, ESTIMATE score, immune score and stromal score in the Risk-H and Risk-L groups. ns, not significant; *, *p *< 0.05; **, *p *< 0.01; *** *p *< 0.001; ****, *p*< 0.0001.

## Data Availability

The datasets generated or analyzed during this study are freely available in the Cancer Genome Atlas (https://tcga-xena-hub.s3.us-east-1.amazonaws.com/download/TCGA.HNSC.sampleMap%2FH iSeqV2_exon.gz). The GSE65858 database was downed from the website as follows, https://www.ncbi.nlm.nih.gov/geo/query/acc.cgi?acc=GSE65858. The GSE67614 dataset was downloaded from website as follows, https://www.ncbi.nlm.nih.gov/geo/query/acc.cgi?acc=GSE67614.

## Abbreviations

HNSCC: Head and neck squamous cell carcinoma
TRRGs: Therapeutic response-related genes
TCGA: The Cancer Genome Atlas
LASSO: Least absolute shrinkage and selection operator
GEO: Gene Expression Omnibus
Risk-H: High-risk group
Risk-L: Low-risk group
OS: Overall survival
AUC: Area under the curve
HPV: Human papillomavirus
HPA: Human Protein Atlas
TME: Tumor microenvironment
CR: Complete response
PR: Partial response
SD: Stable disease
PD: Progressive disease
GO: Gene Ontology
KEGG: Kyoto Encyclopedia of Genes and Genomes
ROC: Receiver operating characteristic
GSEA: Gene set enrichment analysis
FC: Fold change
TMB: Tumor mutational burden
ICB: Immune checkpoint blockade
NK: Natural killer
Tfh: T follicular helper
Tregs: Regulatory T cells
ICI: Immune checkpoint inhibitor
TIME: Tumor immune microenvironment
