# Supplementary figures and images for: Identification of an individualized therapy prognostic signature for head and neck squamous cell carcinoma
Source: BMC Genomics. 2023 Apr 28;24:221. doi: 10.1186/s12864-023-09325-1 (PMC10142243; doi:10.1186/s12864-023-09325-1)

A

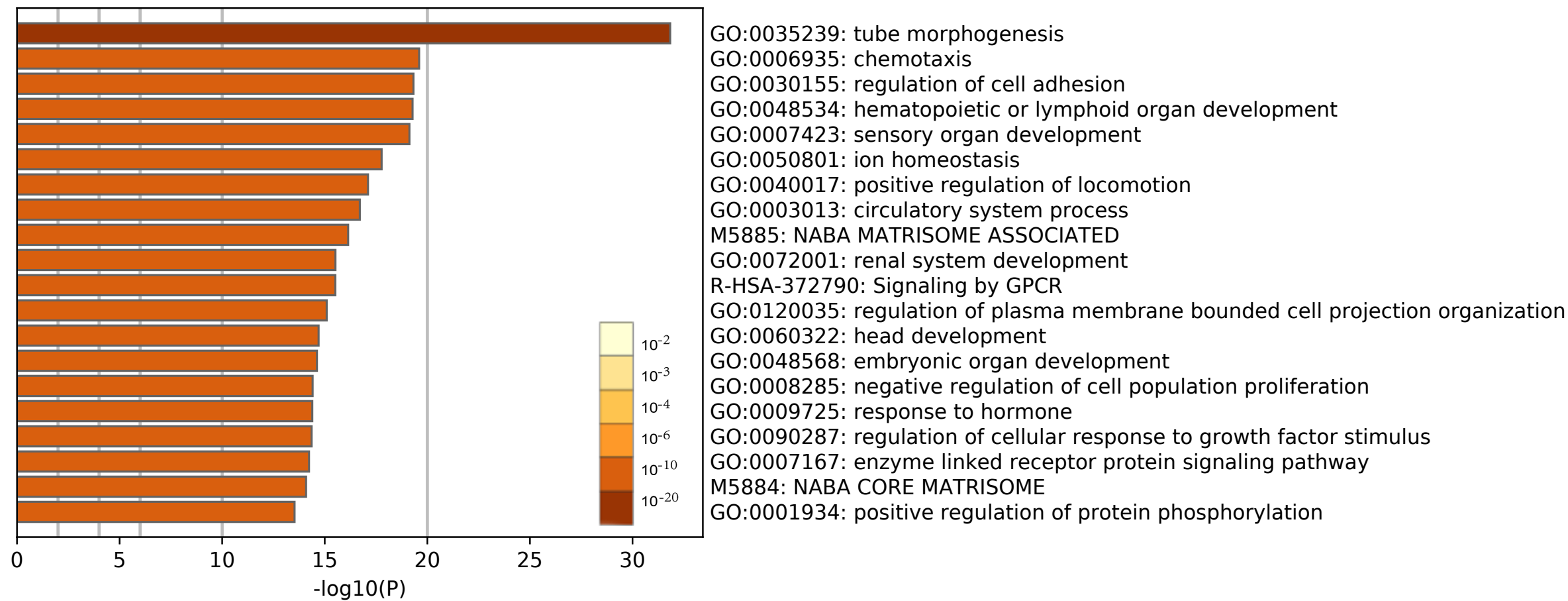

B

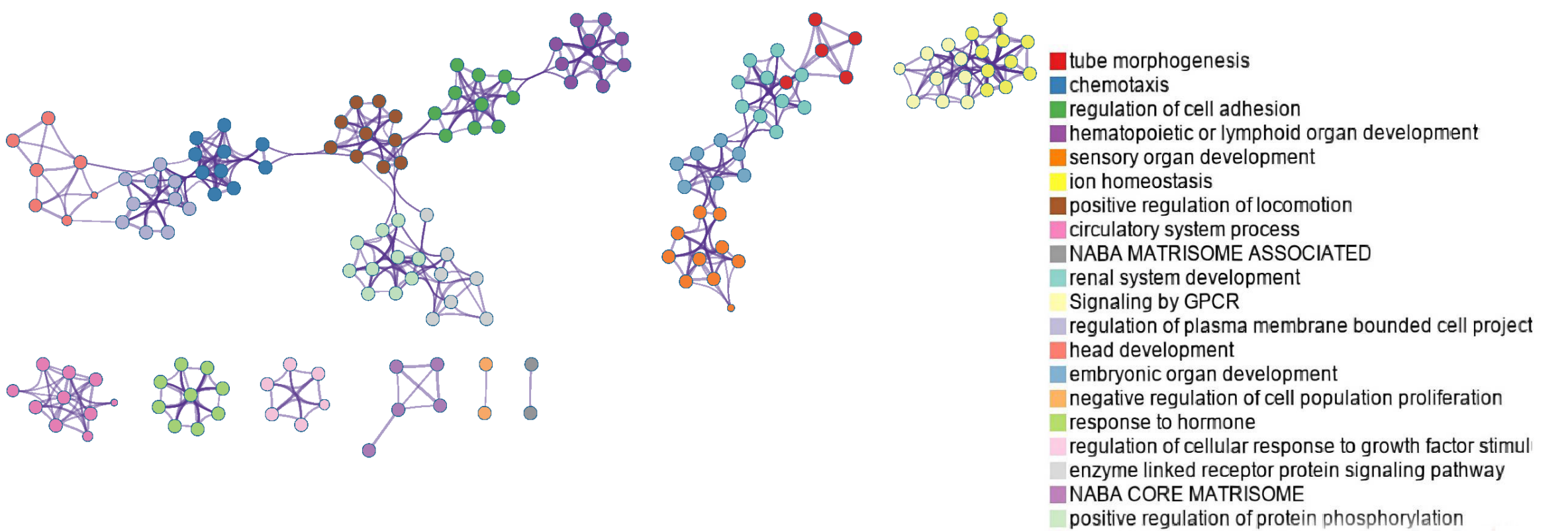

Supplement: Supplementary file 6 — Additional file 6: Fig. S1. Functional enrichment and network analysis in the TCGA-HNSC dataset. A Heatmap of the top 20 functional enrichment terms by Gene Ontology (GO) and Kyoto Encyclopedia of Genes and Genomes (KEGG) analysis based on differentially expressed therapeutic response-related genes (TRRGs). B Protein-protein interaction subnetworks of 20 functional enrichment terms. [file 12864_2023_9325_MOESM6_ESM.pdf]

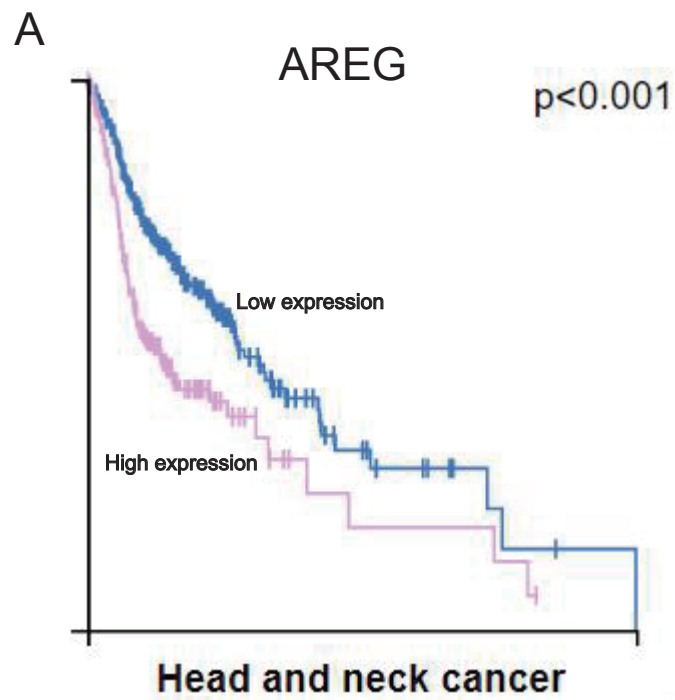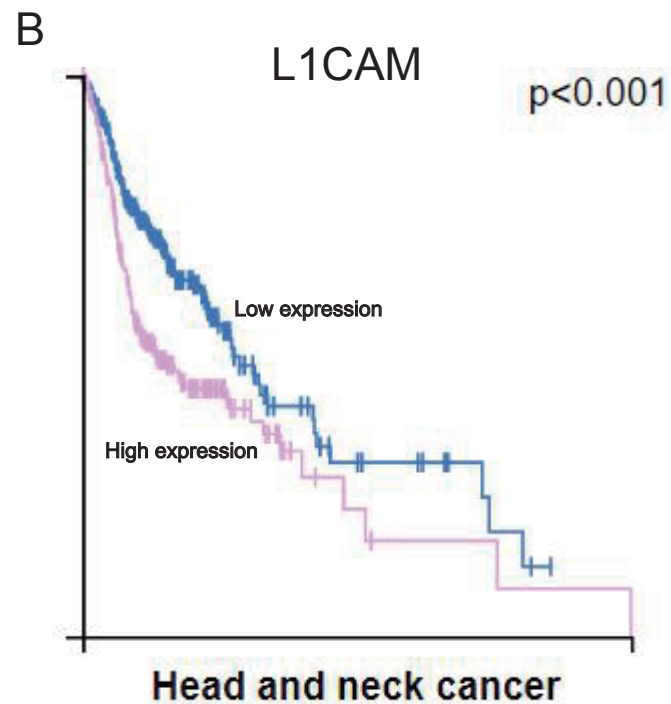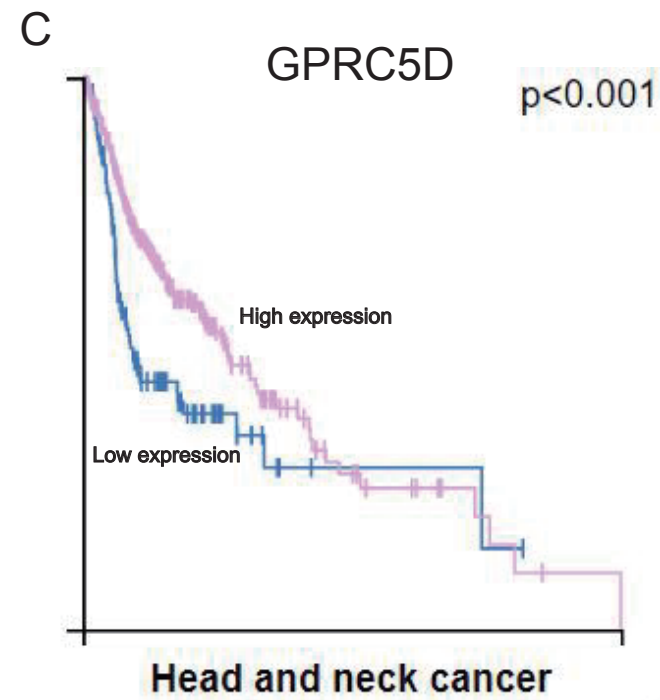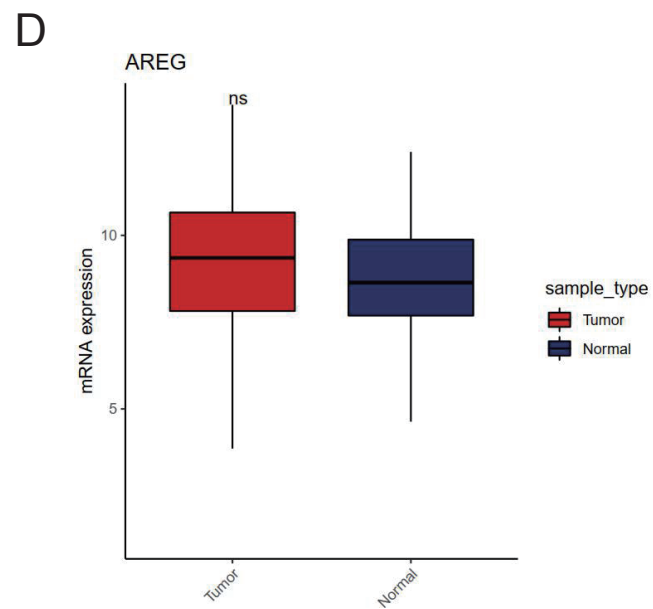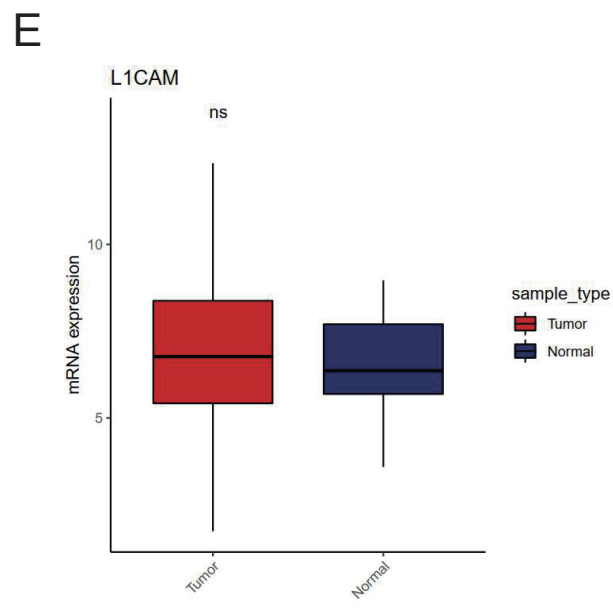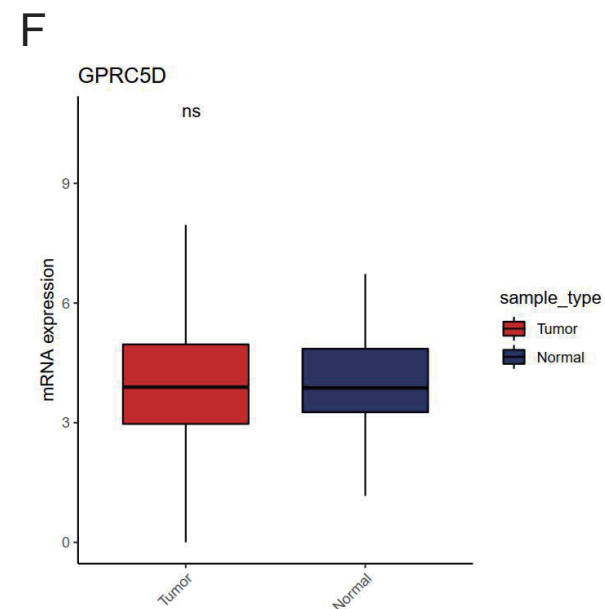

Supplement: Supplementary file 7 — Additional file 7: Fig. S2 Expression information of AREG, L1CAM and GPRC5D. A-C Kaplan Meier analysis showed that AREG, L1CAM and GPRC5D had a good prognostic efficacy in the Human Protein Atlas (HPA) database. D-F mRNA expression of AREG, L1CAM and GPRC5D was not significantly different between cancer and normal tissues in the TCGA database. [file 12864_2023_9325_MOESM7_ESM.pdf]

A

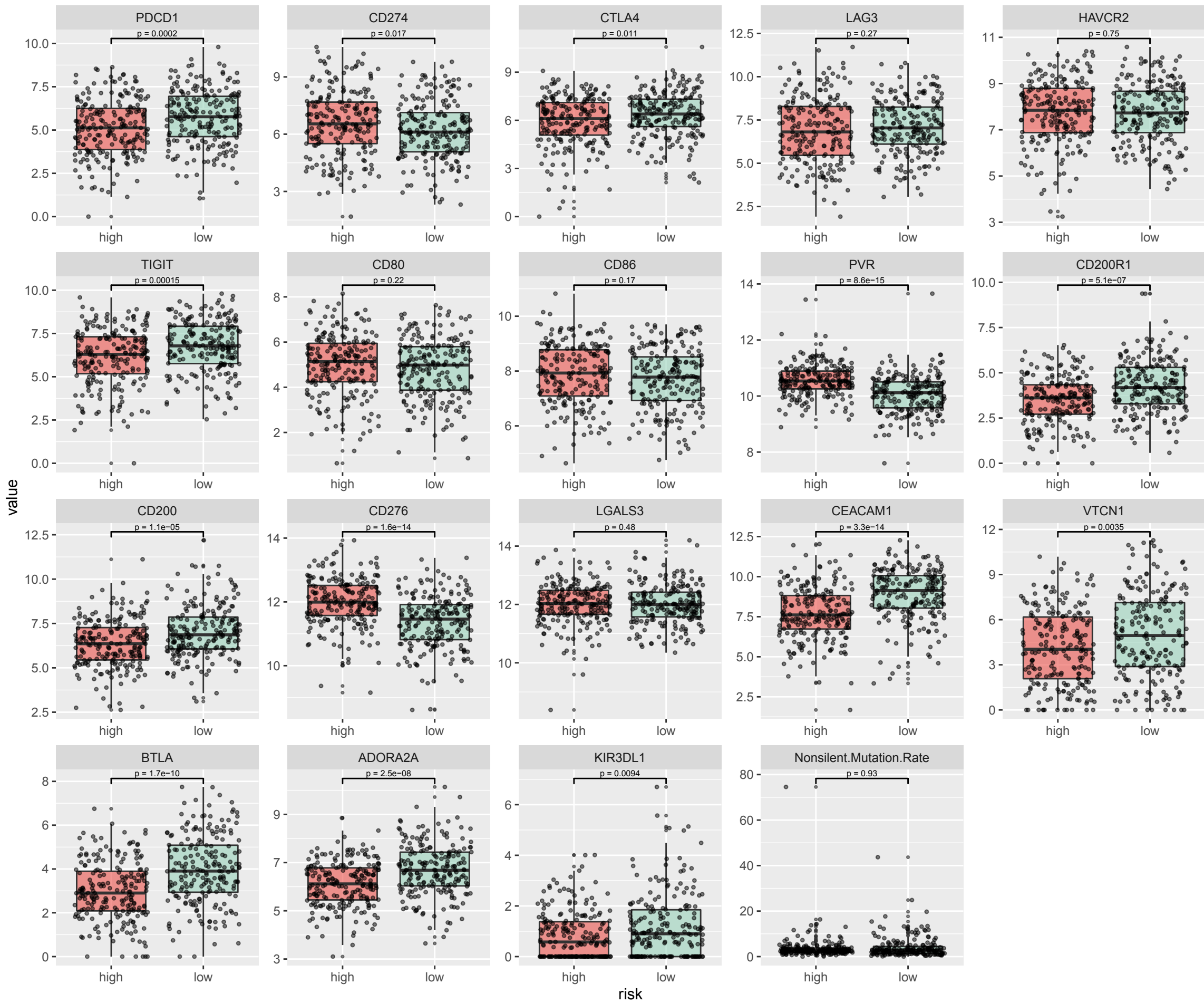

B

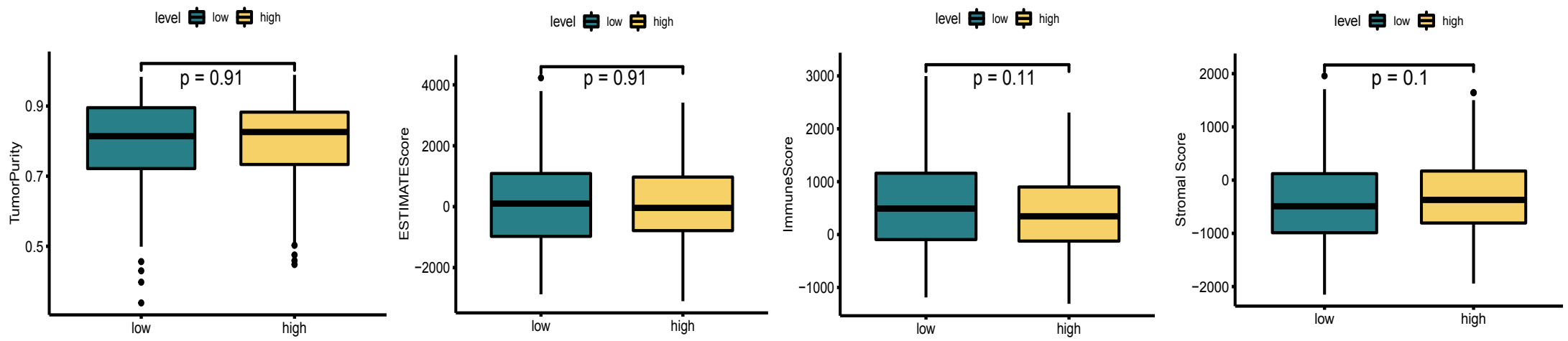

Supplement: Supplementary file 8 — Additional file 8: Fig. S3. Relationships between the risk score and immune checkpoint blockade (ICB)-associated genes and mutation rate. A ICB-associated genes and nonsilent mutation rate in the Risk-H and Risk-L groups. B Tumor purity, ESTIMATE score, immune score and stromal score in the Risk-H and Risk-L groups. ns, not significant; *, p < 0.05; **, p < 0.01; *** p < 0.001; ****, p< 0.0001. [file 12864_2023_9325_MOESM8_ESM.pdf]
